# Comparing TCIM use and patient satisfaction in germany: impact of health self-management across migration backgrounds

**DOI:** 10.1186/s41043-026-01395-7

**Published:** 2026-07-17

**Authors:** Steffen Grüll, Jutta Huebner, Gianluca Ciarlo

**Affiliations:** 1 Jena University Hospital, Am Klinikum 1, Jena, 07747 Germany; 2 Medizinische Klinik I, Universitätsklinikum Frankfurt, Theodor-Stern-Kai 7, Frankfurt am Main, 60596 Germany; 3 Department of Gastroenterology, hepatology, pneumology, allergology, nutritional medicine, endocrinology, University Hospital Frankfurt, Theodor-Stern-Kai 7, Frankfurt am Main, 60590 Germany

**Keywords:** TCIM use, Patient satisfaction, Health self-management, Migrant health

## Abstract

**Purpose:**

Cultural background and migration experiences shape health behavior, use of traditional, complementary and integrative medicine (TCIM), and trust in physicians. This study examines how these factors interact in patients in Germany and whether health self-management modifies these relationships. The goal is to inform culturally sensitive, patient-centered care.

**Methods:**

Between October and December 2023, 375 patients were surveyed in a monocentric primary care study using an anonymous questionnaire assessing migration background, use of TCIM, health self-management, trust in physicians, and relevant sociodemographic characteristics.

**Results:**

A total of 375 patients participated (mean age 50.3 years, 54.5% female), with 19.5% reporting a migration background. Overall patient activation was moderate to high (M = 2.86–3.66, SD = 0.55–0.92), with no significant differences between natives and migrants (Cohen’s d = 0.067, Hedges’ g = 0.067, Glass’ Δ = 0.050). Physical exercise (75.2%), other vitamins/dietary supplements (40.5%), and relaxation techniques (10.9%) were the most frequently used TCIM methods, while mistletoe (1.1%), probiotics (6.1%), acupuncture/acupressure (3.2%), and Reiki (3.2%) were rarely applied. Patient satisfaction was high across all participants (mean range 5.42–6.08 on a 7-point Likert scale), with natives reporting slightly higher scores for general satisfaction and trust in physician competence.

**Conclusion:**

Patient activation was moderate to high, with no significant differences between native-born and migrant participants. Lifestyle-oriented TCIM methods were used most frequently, with little difference in usage. Overall patient satisfaction was high, although migrants reported slightly lower confidence and satisfaction. These findings underscore the need for culturally sensitive measures to promote equitable healthcare.

## Introduction

Recognizing patients’ individual needs is a key foundation for interacting with them and for the physician-patient relationship. Migration experiences and cultural influences can have a significant impact on communication styles, decision-making processes, and trust in medical institutions [1]. Cultural norms and family structures may influence how treatment decisions are made and the extent to which patients and relatives are involved in the decision-making process [2]. In addition, different experiences with the healthcare system shape expectations, attitudes, and the use of medical services, as well as the relationship with the treating physician [3–4].

Empirical studies suggest that the degree of patient satisfaction can vary significantly between patients with and without a migration background. These differences appear to be significantly influenced by the congruence between patients’ individual preferences and the care services actually provided [5–6]. In addition, experiences with and access to conventional healthcare may differ from those related to traditional healing practices [7]. This highlights the particular importance of a culturally sensitive approach in health management and counseling.

Sociodemographic factors are key determinants of health behavior and the use of traditional, complementary and integrative medicine (TCIM). Characteristics such as age, gender, educational level, income, and occupational status influence both the perception of health and the willingness to use relevant services [8–9]. Empirical findings show, for example, that people with higher levels of education are more likely to use TCIM, while socioeconomically disadvantaged groups have less access to such services [8]. In addition, cultural and linguistic backgrounds influence the choice of treatment methods, expectations of medical care, and trust in health information [10–11]. It is therefore essential to take these variables into account in order to understand differences in health behavior and develop targeted care strategies.

Evidence from high-income countries with universal or well-established healthcare systems suggests that frequent users of TCIM may, in some settings, report higher satisfaction with medical care [12]. Using these services can strengthen feelings of self-efficacy and control over one’s own health, while also promoting trust in the healthcare system. At the same time, individual self-management influences how patients seek, understand, and apply health information. More pronounced self-management often correlates with higher satisfaction, more active use of preventive measures, and increased use of TCIM services [13]. In addition, self-management can moderate differences in treatment success and perceived quality of care [14–15]. Social, cultural, and individual factors shape the nature of self-management, the promotion of which is a key prerequisite for patient-centered care and empowerment [16].

This study examines how migration experiences and cultural influences of patients with a migration background in Germany affect their use of TCIM services and their trust in physicians. It also analyzes whether self-management reinforces, weakens, or mediates these relationships. The aim is to identify key factors that are important for culturally sensitive and patient-centered care and effective physician-patient communication.

## Patients and methods

### Study population

This monocentric, prospective cross-sectional study was conducted in an outpatient primary care practice that provides annual medical care to approximately 4,000 patients and serves a large and diverse migrant population. Between October and December 2023, 375 patients were recruited using a standardized, anonymized questionnaire assessing sociodemographic characteristics, and patient activation, trust in physicians, satisfaction with medical care, and the use of TCIM.

All participants received written information prior to enrollment and provided informed consent. Recruitment was carried out on site by trained clinical staff to ensure consistent administration of study materials.

The high cultural and linguistic diversity of the patient population offers a robust empirical basis for examining the interplay between acculturation processes, resilience, and trust in healthcare providers.

### Inclusion and exclusion criteria

Participants were eligible if they were able to provide informed consent and had sufficient language proficiency to complete the questionnaire independently or with the support of an interpreter. Patients who did not meet these criteria were excluded.

### Endpoints

The primary endpoints of this study include the extent of TCIM use and the degree of patient satisfaction and patient activation among individuals with and without a migration background. The analysis examines how these factors relate to one another and whether health self-management modifies these associations. Secondary endpoints include cultural influences and patient preferences that may shape TCIM use, satisfaction, and activation, as well as differences between patients with and without a migration background. Sociodemographic variables are additionally considered to identify further determinants of health behavior and to derive implications for patient education on TCIM use, particularly for migrant populations.

### Questionnaire

A standardized questionnaire was developed to comprehensively assess the interplay between traditional, complementary and integrative medicine (TCIM) use, patient satisfaction, patient activation, and health self-management among patients with and without a migration background. The questionnaire was based on a literature review of previously validated instruments. To assess patient activation, the German version of the 13-item Patient Activation Measure (PAM-13D) was applied [17–18]. Prior to full deployment, the questionnaire underwent a pilot test with 20 patients to ensure clarity, feasibility, and appropriateness of items. The questionnaire encompassed five interrelated domains: (1) sociodemographic characteristics and health status, (2) health-related attitudes and self-management, (3) trust in physicians and satisfaction with healthcare, (4) spiritual beliefs and practices, and (5) resilience-related capacities. This multidimensional instrument was designed to enable nuanced profiling of psychosocial and cultural factors influencing healthcare engagement in a primary care population with a high proportion of migrants.

#### Sociodemographic and Health Characteristics:

Participants provided information on their age, gender, country of birth, length of stay in Germany, place of residence, religion, level of education, and previous or current illnesses. Closed response formats with optional free text fields enabled standardized data collection while also capturing individual details. For participants with a migration background, additional migration- and culture-related characteristics were recorded, including estimated German language skills, self-assessed health, and language use in various contexts such as reading, speaking, thinking, media use, at home, and in social situations. In addition, social preferences regarding the cultural background of friendships, social contacts, and children’s friendships were documented.

#### Health-Related Attitudes and Self-Management:

This domain included items assessing personal responsibility for health, perceived ability to prevent or manage illness, knowledge of medications and treatment options, confidence in implementing lifestyle changes, and maintenance of health-promoting behaviors. Responses were rated on a four-point Likert scale from “not true” to “true,” capturing participants’ perceived self-efficacy and engagement in health management.

**Trust in Physicians and Satisfaction with Healthcare**: Trust was evaluated through statements addressing confidence in physicians’ decisions, perceived thoroughness and reliability, and overall trust in medical professionals. Satisfaction with the healthcare system was assessed using items on communication quality, perceived attention, and patient-centeredness, rated on a seven-point Likert scale from “strongly disagree” to “strongly agree.” Total satisfaction scores were interpreted as follows: 10–24 points = low satisfaction, 25–39 points = moderate satisfaction, 40–54 points = high satisfaction, and 55–70 points = very high satisfaction.

#### Spiritual Beliefs and Practices:

Items assessed the importance of prayer and personal reflection, perception of divine presence, and religious coping as a source of comfort and guidance. Responses were collected using four-point Likert scales ranging from “does not apply” to “fully applies,” providing insight into existential and spiritual resources.

#### Resilience:

Resilience-related capacities were measured via self-assessment of adaptive coping, stress management, persistence in the face of adversity, flexibility, and maintenance of emotional stability. Items were rated on five-point Likert scales from “not true at all” to “always true,” capturing participants’ perceived ability to recover from stressors and maintain psychological well-being.

#### Traditional, complementary and integrative medicine (TCIM) Use and Media Trust:

TCIM use was assessed using dichotomous items (yes/no) based on the German version of the International Complementary and Alternative Medicine Questionnaire (I-CAM-G) [19]. Assessed modalities included vitamin and micronutrient supplementation, herbal remedies, mistletoe therapy, acupuncture/acupressure, yoga, Tai-Chi, Qi Gong, physical activity, specific dietary approaches (e.g., ketogenic diet, vegan diet, fasting), and relaxation techniques such as meditation, mindfulness exercises, autogenic training, and progressive muscle relaxation. Participants could additionally report further practices in open-ended responses. Trust in different information sources, including media and social media, was evaluated on a seven-point scale ranging from “no trust at all” to “very high trust.” This comprehensive instrument allowed the examination of interrelations between TCIM use, patient satisfaction, patient activation, health self-management, cultural influences, and patient preferences among a culturally diverse primary care population.

### Statistical analysis

Statistical analyses were conducted using IBM SPSS Statistics (Version 29). Descriptive statistics, including frequencies, percentages, means, standard deviations, and ranges, were calculated to characterize the study population and summarize key variables, such as acculturation, resilience, and trust in medical care. Group differences, for instance between participants with varying levels of acculturation, were examined using independent-samples t tests for continuous variables and chi-square tests for categorical variables. Homogeneity of variances was assessed using Levene’s test. To complement significance testing, effect sizes were calculated using Cohen’s d, Hedges’ g, and Glass’ Δ. These measures were included to provide a more comprehensive assessment of the magnitude of differences between natives and migrants, thereby allowing interpretation beyond statistical significance.

To evaluate the associations between acculturation, resilience, and trust in medical care, multivariate linear regression analyses were performed. Model assumptions, overall significance, regression coefficients, and 95% confidence intervals were assessed to determine the strength and reliability of the observed relationships. All analyses were interpreted within the context of exploratory research, given the cross-sectional design and the observational nature of the study.

### Ethical vote

This research adhered to the ethical standards of the Declaration of Helsinki. Participation was voluntary and anonymous. By returning the completed questionnaire, participants provided implied informed consent. Ethical approval was granted through two separate applications: by the Ethics Committee of the University Hospital Jena and by the Ethics Committee of the State Medical Association of Baden-Württemberg, the latter required because data collection took place in a rural general practice in Neudenau near Heilbronn. The study received approval on October 16, 2023 (Approval No. 2023-3129-Bef).

## Results

### Demographic data

Overall, between October and December 2023, a total of 375 patients were included. All participants provided their age, with a mean of 50.3 years. The youngest participant was 18 years old, and the oldest was 88 years. Regarding gender distribution, 54.5% of participants were female, 45.2% were male, and 0.3% identified as non-binary/diverse. Table 1 summarizes the analyses of the demographic data.

**Table 1:** demographic data

Category	Variable	*N* [= 375] (%)
Age	Mean [years] ± SD	50,30 ± 16,16
	Minimum [years]	18
	Maximum [years]	88
Gender	Female	204 (54.5)
	Male	169 (45.2)
	Divers	1 (0.3)
	No answer	1
Migration background	Migrants	73 (19.5)
	Natives	302 (80.5)
Marital status	Single	63 (16.9)
	Married	233 (62.5)
	In a partnership / Cohabiting	38 (10.2)
	Divorced	30 (8)
	Widowed	9 (2.4)
	No answer	2
Residence	Metropolis	14 (3.8)
	Rural area	353 (96.2)
	No answer	8
Religion	Christian	265 (71.2)
	Muslim	41 (11)
	Atheist / no religion	62 (16.7)
	Other	4 (1.1)
	No answer	3
Educational background	No school diploma/ graduation	3 (0.8)
	Lower secondary school (Hauptschule, Germany)	141 (39)
	Intermediate secondary school (Realschule, Germany)	129 (35.6)
	General university entrance qualification (Abitur, Germany) / University of applied sciences entrance qualification (Fachhochschulreife, Germany)	46 (12.7)
	University degree	43 (11.9)
	No answer	13
Number of children	No children	104 (27.8)
	One	64 (17.1)
	Two	134 (35.8)
	More than two children	72 (19.3)
	No answer	1
Chronic disease	None	180 (49.6)
	≥ 1^1^	183 (50.4)
	No answer	12

SD= standard deviation, n= number, ^1^chronic disease= Asthma, cardiac arrhythmia, and diabetes mellitus

### Overview of patients with a migrant background

Table 2 summarizes the demographic characteristics of participants with a migration background. Overall, 19.5% of respondents reported a migration background. According to the study definition, this category also included second-generation migrants. Among these participants, 47.9% were born in Germany, while 21.1% were born in Turkey and 8.2% in Poland. Overall, individuals with a migration background represented 15 different countries of origin. Most migrants reported good or excellent German skills and health, and the majority (80.8%) had lived in Germany for over 20 years.

**Table 2:** Summary of demographic data on patients with a migration background

Category	Variable	*N* [= 73] (%)
Country of birth (including second-generation migrants)^1^	Germany	34 (47.9)
	Turkey	15 (21.1)
	Poland	6 (8.5)
	Hungary / Lithuania / Pakistan / Romania^2^	8 (11.3)
	Bosnia-Herzegovina / Brasil / Croatia / Italy / Kazakhstan / Kyrgyzstan / Serbia / USA^3^	8 (11.3)
	No answer	2
Knowledge of German	Excellent	24 (33.3)
	Very good	16 (22.2)
	Good	19 (26.4)
	Sufficient	10 (13.9)
	Poor	3 (4.2)
	No answer	1
General state of health	Excellent	3 (4.2)
	Very good	10 (14.1)
	Good	38 (53.5)
	Sufficient	16 (23)
	Poor	4 (5.6)
	No answer	2
Resides in Germany [in years]	1–5	1 (1.4)
	5–10	4 (5.5)
	10–20	9 (12.3)
	≥ 20	59 (80.8)

Notes: ^1^= Participants born in Germany were classified as having a migration background if at least one parent had migrated to Germany, ^2^= two patients per country, ^3^= one patient per country. Percentage may not sum to 100% due to rounding

### Patient activation among natives and migrants

The descriptive analyses show moderate to high levels of patient activation in the sample overall (M = 2.86–3.66; SD = 0.55–0.92). Particularly high values were observed for items reflecting personal responsibility and active involvement in healthcare, such as taking responsibility for one’s own health (M = 3.66, SD = 0.55, 95% CI [3.61; 3.72]). Lower mean values and greater variation, on the other hand, occur in more complex knowledge and self-management requirements, such as understanding different treatment options (M = 2.86, SD = 0.92, 95% CI [2.77; 2.96]). Overall, the data indicate a solid awareness of prevention among participants, coupled with uncertainties in more demanding health-related tasks.

The calculated effect sizes for testing group differences between natives and migrants do not indicate any significant differences in patient activation. Cohen’s d yielded a very small effect size of 0.067 (95% CI [-0.192; 0.326]), Hedges’ g showed an identical point estimate with an almost identical confidence interval (0.067; 95% CI [-0.191; 0.325]), and Glass’ Δ also showed a very small effect size of 0.050 (95% CI [-0.209; 0.309]). All confidence intervals overlap the zero point, indicating that there is no statistically or practically relevant difference in patient activation between natives and migrants.

### TCIM use in a socioculturally heterogeneous population

**Fig. 1:**
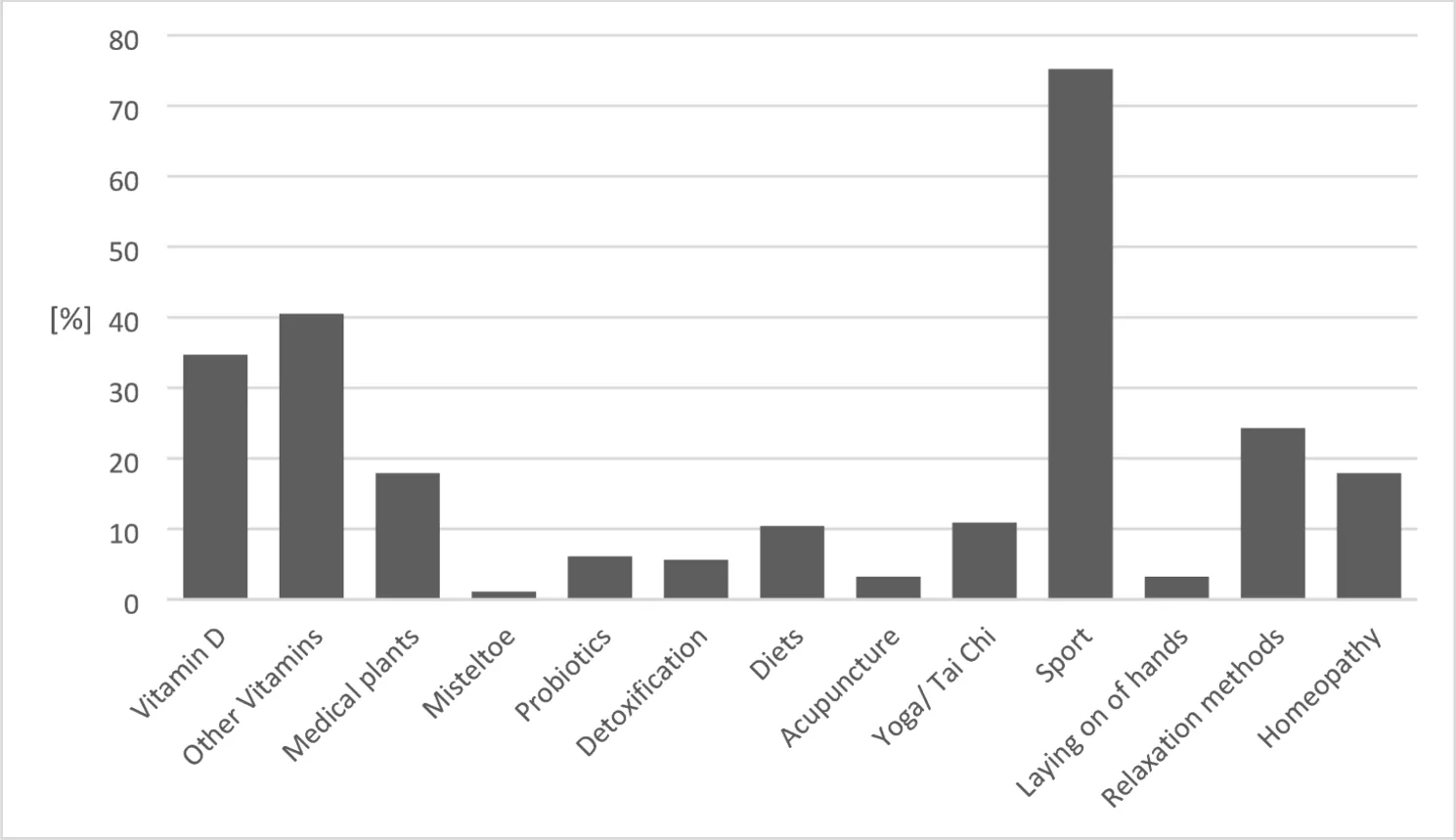
Overview of TCIM methods

Among the surveyed patients, physical exercise was the most frequently used traditional, complementary and integrative medicine (TCIM) method (75.2%), followed by other vitamins and dietary supplements (40.5%) and relaxation techniques (10.9%), while therapies such as mistletoe (1.1%), probiotics (6.1%), acupuncture/acupressure (3.2%), and Reiki/laying on of hands (3.2%) were rarely applied. Figure 1 provides an overview of the TCIM methods utilized by the participants. Reliability analysis of the TCIM questionnaire showed moderate internal consistency (Cronbach’s α = 0.654–0.713 after item removal), reflecting substantial variability in responses. Logistic regression analysis indicated that most TCIM therapies did not significantly differ between natives and migrants, with p-values well above 0.05 for the majority of interventions. However, notable exceptions were observed for other vitamins/supplements (*p* = 0.041, OR = 0.511), physical exercise (*p* = 0.033, OR = 2.043), and homeopathy (*p* = 0.007, OR = 5.156), indicating significant differences in utilization patterns between natives and migrants. In particular, native participants reported more frequent use of homeopathy.

### Healthcare satisfaction: native and migrant patients

Patient satisfaction was assessed using ten items on a 7-point Likert scale. The mean values ranged between 5.42 and 6.08, with particularly high values for “I have confidence in my doctor’s knowledge and skills” (mean = 6.08) and “My doctor listens to me attentively” (mean = 6.02). The median and mode were consistently 6, reflecting a consistently high level of agreement.

The separate analysis for natives and migrants revealed generally high satisfaction scores in both groups. Mean values were comparable across most items, with only minor numerical differences and similar variability. For the majority of statements, these differences did not reach statistical significance.

Independent-samples t tests and Levene’s tests confirmed this pattern: there were no significant differences between the groups for most statements. However, significant differences were found for “I am satisfied with how my doctor took care of me” (t = 2.079; *p* = 0.038; mean difference = 0.312) and “I have confidence in my doctor’s knowledge and skills” (t = 2.121; *p* = 0.036; mean difference = 0.265). For all other statements, the p-values were above 0.05, meaning that there were no statistically relevant differences.

Both groups rated medical care very positively overall. Significant differences only existed in terms of general satisfaction with care and trust in the physician’s competence, with native participants reporting higher ratings. The effect sizes of these differences are small to medium.

## Discussion

This study included 375 patients and reflected a middle-aged population with a balanced gender distribution and a migrant population of 19.5%, most of whom were long-term residents and had a good command of German. Descriptive analyses showed generally high patient activation and very small, non-significant differences between native-born and migrant patients, suggesting comparable levels of self-management confidence across all groups. The use of TCIM was dominated by lifestyle-oriented methods, and although most TCIM practices did not differ significantly by migration status, there were notable differences in dietary supplements, physical activity, and homeopathy. Patient satisfaction with medical care was high in both groups, with only minor differences in favor of the native population in terms of overall satisfaction and trust in the competence of physicians.

With respect to patient activation, both natives and migrants demonstrated a strong sense of responsibility and motivation to take proactive health measures. Descriptively, migrants showed greater uncertainty such as independently dealing with treatment options or carrying out medical measures. Migrants with low local language skills show significantly reduced use of internet-based health information and are less able to research alternative treatment options or make therapy decisions [20]. A lack of culturally sensitive, understandable, and accessible information leads to gaps in knowledge regarding the necessity of medication, lifestyle interventions, and the correct implementation of medical measures [21]. This results in uncertainty, low adherence, and suboptimal self-management. Migrants therefore resort more frequently to informal networks, lay help, or self-medication, which increases the risk of misuse and complications [22].

Although overall patient activation was comparable between groups, native participants showed a tendency toward greater confidence in dealing with complex health decisions and more frequent use of certain TCIM approaches [23–25]. The higher use of homeopathy among native participants may partly reflect characteristics of the German healthcare and cultural context. Given the long tradition and continued acceptance of homeopathy in Germany, utilization patterns are likely shaped not only by migration-related cultural factors, but also by the norms and practices of the host country. In addition, differences in health literacy, language barriers, and access to health-related information may further contribute to these patterns [20, 26]. These findings underscore the importance of interventions aimed at strengthening health literacy, practical implementation skills, and culturally sensitive care in order to support sustainable self-management, particularly among migrants.

Differences in the use of TCIM, rather than overall patient activation, were observed between native-born individuals and migrants. In agreement with the Multiethnic Cohort Study and other European studies, it has been shown that the use of dietary supplements and vitamins varies according to migration status and ethnicity [27–29]; natives use these supplements more often than migrants, which is influenced by socioeconomic factors and health literacy [8]. In our sample, physical activity, dietary supplements, and relaxation techniques were among the most commonly used TCIM methods, which is consistent with findings from population-based studies in which lifestyle-oriented interventions are used more frequently than biologically or spiritually oriented methods [30–31]. These results are comparable to findings from international studies, which indicate that cultural preferences, access to information, socioeconomic factors, and trust in certain therapies significantly influence the use of TCIM [31–32]. Overall, this shows that intervention approaches designed to promote patient activation and TCIM use should focus more on education, practical implementation skills, and cultural sensitivity, especially among migrants, in order to reduce existing differences and support sustainable health-promoting behaviors.

At the same time, structural aspects of the healthcare system also shape these usage patterns. Studies indicate that physicians often collect insufficient information on patients’ TCIM use [33], and limited knowledge as well as inadequate training in this field further restrict patients’ access to evidence-based guidance. This lack of systematic inquiry and professional knowledge can contribute to differences in TCIM use, particularly among groups with lower health literacy. In line with this, Krug et al. [34] report that patients with chronic or mental illnesses increasingly turn to alternative sources of information, such as alternative practitioners, when seeking advice on TCIM.

A significant correlation between the quality of physician-patient communication and patient satisfaction has been established in studies. High satisfaction is promoted by clear communication of information and adequate communication skills [35]. Language barriers, on the other hand, appear to produce the opposite result. As our data show, patients with a migration background who subjectively feel a lack of appreciation or respect have lower satisfaction rates. Several studies show that experiences of discrimination, the feeling of not being taken seriously, and a lack of consideration for cultural backgrounds lead to mistrust of medical staff and a negative assessment of care [36]. This leads to a lower willingness to follow medical recommendations or attend follow-up examinations [37]. When such language barriers are overcome, other aspects, such as attentively listening, empathy, and involvement in treatment decisions [38], come to the fore and trust in the physician´s abilities increases. Topçu & Mösko [39] conclude that barrier-free communication not only leads to greater satisfaction, but also makes patients more willing to share confidential information.

Several recommendations for action can be derived from the results for dealing with TCIM use among migrants. Healthcare professionals in conventional healthcare settings should receive additional training in TCIM, with particular attention to cultural differences in the use of specific therapies. In addition, patient activation should be maintained or further strengthened through appropriate chronic disease management programs, ideally through multilingual services to reduce language barriers. In addition, the expansion of interpreting services and culturally sensitive training for medical staff are necessary to better meet the special needs of migrants. Overall, these measures can help to ensure and further improve high patient satisfaction.

### Limitations and future directions

This study has several limitations. First, it was conducted in a single practice, resulting in a selective patient population, and voluntary participation posed a challenge, particularly in recruiting migrant patients due to concerns about the questionnaire and its length. Second, the literature search was limited to English- and German-language sources within a limited time frame, which may have excluded relevant studies in other languages or from other time periods. Furthermore, most of the participating migrants had been living in Germany for over 20 years, suggesting a high degree of integration and familiarity with the healthcare system, which may limit the generalizability of the results to less integrated migrant groups. Despite these limitations, the study successfully replicates many findings from previous research and provides valuable insights into patient activation, the use of complementary medicine, and satisfaction with health care.

## Conclusion

The findings suggest that patient activation is largely comparable among native-born individuals and immigrants, indicating that long-term immigrants can achieve similar levels of self-management confidence and health engagement as the native-born population. The observed differences in the use of certain TCIM methods, such as homeopathy, physical exercise, and dietary supplements, point to cultural, informational, and possibly socioeconomic influences on health behavior and underscore the need for tailored education and intervention strategies. The overall high patient satisfaction in both groups shows that the health care system meets basic expectations. However, the slightly lower satisfaction and trust scores among migrants indicate that there is room for further improvement in culturally sensitive communication and support. Taken together, these findings underscore the importance of developing interventions that combine patient activation, culturally adapted TCIM counseling, and effective physician-patient communication to promote equitable and sustainable health outcomes in diverse populations.

## Data Availability

The dataset supporting the conclusions of this article is included within the article.
